# Small Colony variants of *Staphylococcus aureus* isolated from a patient with infective endocarditis: a case report and review of the literature

**Published:** 2012-06

**Authors:** S Bhattacharyya, S Roy, PK Mukhopadhyay, K Rit, JB Dey, U Ganguly, R Ray

**Affiliations:** Department of Microbiology, Bankura Sammilani Medical College, Bankura, West Bengal, India

**Keywords:** Small colony variant, *Staphylococcus aureus*, Menadione, Auxotrophicity

## Abstract

*Staphylococcus aureus* produces a particular morphological variant called small colony variant (SCV) which is responsible for persistent subclinical infections in predisposed individuals and is usually resistant to aminoglycosides and cell wall active antibiotics. Infections by SCV of *S. aureus* are an upcoming problem due to difficulty in laboratory diagnosis and resistance to antimicrobial chemotherapy. We here report a case of infective endocarditis caused by SCV of *Staphylococcus aureus* in a pediatric patient.

## INTRODUCTION

*Staphylococcus aureus* can cause a variety of infections like skin and subcutaneous infections, pneumonia, osteomyelitis, toxic shock syndrome and infective endocarditis (1). It also produces a particular variant called small colony variant (SCV) which is responsible for persistent subclinical infections and is usually resistant to aminoglycosides and cell wall active antibiotics. Infections by SCV of *S. aureus* is an emerging problem owing to difficulty in diagnosis due to atypical phenotypic characteristics and refractoriness to therapy. We here report a case of infective endocarditis due to SCV of *Staphylococcus aureus* in a pediatric patient.

**Case report**. J, a 3 1/2 year old female patient, was admitted in the pediatric ward of BSMC, Bankura on 14.8.2010 with symptoms of respiratory distress, swelling of the legs and fever. There was no cyanosis. Chest auscultation showed pansystolic precordial murmur. Echocardiography was carried out which revealed a subaortic VSD (Ventricular Septal Defect). Blood was sent for bacterial culture and patient was administered Ceftriaxone and Amikacin intramuscularly (i.m.). A few days later, i.m. injection of Ofloxacin was added to the drug regime. Blood culture on nutrient broth with 1% glucose turned turbid after 3 days of aerobic incubation at 37°C. Subculture from the liquid medium on 5% Sheep Blood agar grew small, (0.1. mm diameter), non-hemolytic colonies following incubation at 37°C. There was no growth on MacConkey Agar. The colonies were subcultured on Nutrient Agar and grew as small, non-pigmented colonies after overnight incubation at 37°C that were catalase positive and oxidase negative. Gram stain revealed Gram positive cocci of variable sizes arranged in clusters. There was no growth in 6.5% NaCl broth at 37°C after 3 days aerobic incubation. Coagulase was positive but delayed, and that too after addition of excess of plasma. Antibiotic susceptibility test showed sensitivity to Tetracycline, Amikacin, Erythromycin, Cotrimoxazole and Ofloxacin and resistance to Oxacillin. Few colonies were incubated in nutrient broth and incubated at 37°C till a turbidity of 0.5. Mc Farland standard was attained. A lawn culture was put up on Nutrient Agar with Vitamin K (Menadione) discs of 20 µg and 40 µg strength. After incubation for 24 hrs at 37°C, large colonies grew around the Menadione discs. These large colonies were catalase and coagulase positive and oxidase negative. Gram stain showed Gram positive cocci in clusters. Thus, auxotrophiocity for Menadione was confirmed.

## DISCUSSION

Small colony variants (SCVs) of *S. aureus* cause persistent, long term infections in patients with long standing predisposing conditions like cystic fibrosis and osteomyelitis (1). They are usually auxotrophic for Vitamin K, Thymidine and Hemin and are hence lacking in essential components of the electron transport chain (2). So drugs like aminoglycosides cannot be transported across the cell membrane due to reduction of transmembrane potential (3). Hence SCVs are resistant to Aminoglycosides and beta-lactams and other antibiotics (3). Their slow growth and defective electron transport chain confers them survival advantage and increased biological fitness inside cells and biofilms (2).They also have reduced α- toxin gene expression and hence cause less damage to host cells (2). Therefore they are notorious for causing persistent, indolent infections.

Diagnosis is usually achieved by reversal to wild type *Staphylococcus aureus* colonies in presence of Vitamin K, resistance to Cotrimoxazole (auxotrophicity for thymidine) and increased size and growth around hemin or factor X discs. SCVs of *S. aureus* are often mistaken for Coagulase negative staphylococci unless proper suspicion is there. Electron microscopy as well as Gram staining shows the SCVs to be slightly irregular in size due to incomplete separation after cell division, which adds to the diagnostic dilemma (4). Proper awareness, suspicion and diagnosis is essential since SCVs are often misidentified. Treatment usually consists of combination of antibiotics with Rifampicin for prolonged periods (5).

There are a few reports of isolation of SCVs from clinical specimens. One report mentions isolation of SCVs from pacemaker related endocarditis from Germany (1). To the best of our knowledge, this is the first report of Small colony variant of *S. aureus* from blood of a patient with VSD having infective endocarditis. SCV morphotype was selected since the patient was on aminoglycoside antibiotic, and there are studies quoting that SCVs are selected from wild type *S. aureus* as soon as 24 hours after initiation of aminoglycoside therapy. It would not be unreasonable to infer that SCV of *S. aureus* was responsible for causing infective endocarditis in the patient. Small biofilms in cardiac vegetations can be missed by routine transthoracic Echocardiographic techniques (6).
